# *In vitro*-analysis of kinematics and intradiscal pressures in cervical arthroplasty versus fusion – A biomechanical study in a sheep model with two semi-constrained prosthesis

**DOI:** 10.1186/s12938-015-0018-4

**Published:** 2015-03-24

**Authors:** Dorothea Daentzer, Bastian Welke, Christof Hurschler, Nathalie Husmann, Christina Jansen, Christian Heinrich Flamme, Berna Ida Richter

**Affiliations:** Orthopedic Department, Hannover Medical School, Diakoniekrankenhaus Annastift gGmbH, Anna-von-Borries-Str. 1-7, 30625 Hannover, Germany; Laboratory for Biomechanics and Biomaterials, Department of Orthopedics, Hannover Medical School, Anna-von-Borries-Str. 1-7, 30625 Hannover, Germany; Department of Radiology, Asklepios Clinic Nord, Standort Heidberg, Tangstedter Landstr. 400, 22417 Hamburg, Germany; Department of Spine Surgery, Klinikum Bad Bramstedt GmbH, Oskar-Alexander-Str. 26, 24576 Bad Bramstedt, Germany; Clinic for Orthopedics and Traumatology, Asklepios Clinic Hamburg, Eißendorfer Pferdeweg 52, 21075 Hamburg, Germany; Aesculap AG, Am Aesculap-Platz, 78532 Tuttlingen, Germany

**Keywords:** Total disc replacement, Cervical spine, Adjacent level biomechanics, Intradiscal pressure, Cervical arthroplasty, Disc prosthesis, Fusion, Arthrodesis, Range of motion, Sheep model

## Abstract

**Background:**

As an alternative technique to arthrodesis of the cervical spine, total disc replacement (TDR) has increasingly been used with the aim of restoration of the physiological function of the treated and adjacent motions segments. The purpose of this experimental study was to analyze the kinematics of the target level as well as of the adjacent segments, and to measure the pressures in the proximal and distal disc after arthrodesis as well as after arthroplasty with two different semi-constrained types of prosthesis.

**Methods:**

Twelve cadaveric ovine cervical spines underwent polysegmental (C2-5) multidirectional flexibility testing with a sensor-guided industrial serial robot. Additionally, pressures were recorded in the proximal and distal disc. The following three conditions were tested: (1) intact specimen, (2) single-level arthrodesis C3/4, (3) single-level TDR C3/4 using the Discover® in the first six specimens and the activ® C in the other six cadavers. Statistical analysis was performed for the total range of motion (ROM), the intervertebral ROM (iROM) and the intradiscal pressures (IDP) to compare both the three different conditions as well as the two disc prosthesis among each other.

**Results:**

The relative iROM in the target level was always lowered after fusion in the three directions of motion. In almost all cases, the relative iROM of the adjacent segments was almost always higher compared to the physiologic condition. After arthroplasty, we found increased relative iROM in the treated level in comparison to intact state in almost all cases, with relative iROM in the adjacent segments observed to be lower in almost all situations. The IDP in both adjacent discs always increased in flexion and extension after arthrodesis. In all but five cases, the IDP in each of the adjacent level was decreased below the values of the intact specimens after TDR. Overall, in none of the analyzed parameters were statistically significantly differences between both types of prostheses investigated.

**Conclusion:**

The results of this biomechanical study indicate that single-level implantation of semi-constrained TDR lead to a certain hypermobility in the treated segments with lowering the ROM in the adjacent levels in almost all situations.

## Background

Cervical disc arthroplasty has become an alternative technique to intervertebral fusion. One reason for this trend is the observation that in clinical studies, patients with a history of cervical arthrodesis seem to have a higher incidence of adjacent segment degeneration [1-3]. Furthermore, in biomechanical investigations, most authors have reported an increase in the segmental range of motion (ROM) and the intradiscal pressure (IDP) in the levels proximal and distal to a simulated mono- or bisegmental arthrodesis [4-13]. In contrast, with arthroplasty, the kinematics in the target segment as well as in the neighboring segments reach near to physiologic state, indicating an almost physiologic restoration of the affected motion segments [4,6-8,14,15].

For *in vitro-*testing, cadaveric specimens from the human spine are often used as a biomechanical model. However, factors limiting the interpretation of results include confounding effects due to variability in age, height, gender, stiffness, bone quality, as well as the grade of degenerative changes inherent in cadaver specimens. In contrast, spinal specimens from some animals are generally easier to obtain and more homogeneous than human cadaver specimens. For this reason, several authors have performed comparative biomechanical investigations with the aim of identifying adequate substitute animal models for the human spine. The ovine cervical spine has for instance been suggested as an accepted substitute for *in vitro*-testing [16-18].

The primary objective of the present study was to quantify changes in operative- and adjacent-level kinematics like total ROM (tROM) and intervertebral ROM (iROM) and the IDP after monosegmental arthroplasty in comparison with those in the intact condition and after a single-level arthrodesis. The secondary objective was to compare the results between two different types of semi-constrained disc prosthesis using the same test set-up. As the goal of both implants is to restore the physiologic function of the motion segment and its kinematics, the purpose of this study was to show, whether these criteria can be fulfilled.

## Methods

### Specimen preparation

Twelve fresh frozen cadaveric cervical spines from full-grown sheep with a minimum age of two years were used in this investigation. Before biomechanical analysis, dual-energy x-ray absorptiometry (DXA) was performed to exclude any specimens with pathologically low bone mineral density (t-score more than −2.5). In preparation for biomechanical testing, the cervical spines were thawed to room temperature and dissected clear of all residual musculature. Care was taken to preserve all ligamentous structures and their attachments, as well as the intervertebral discs and facet joint capsules. Three motion segments, from C2 through C5, were tested in a polysegmental setup. The proximal (C2) and distal (C5) ends of the specimen were embedded in cold curing methylmethacrylate resin (Technovit, Heraeus Kulzer GmbH, Wehrheim, Germany) using three perpendicular screws per endplate for additional stabilization. Markers for the optical tracking system were inserted in the vertebral bodies of C2, C3 and C4 by connecting rods. Pressure sensors were placed in the centers of the discs C2/3 and C4/5 under fluoroscopic control.

### Three-dimensional motion segment testing

Testing was performed using a sensor-guided six axis industrial robot (KUKA GmbH, Augsburg, Germany) which is a validated method against the Pure Moment Apparatus (PMA) and capable of applying pure, unconstrained rotational moments about three axes (*x*, *y*, *z*) [19,20]. Hereby, nondestructive pure-moment loading modes were applied: flexion and extension (*x*-axis, ±2.0 Nm), lateral bending (*z*-axis, ±2.0 Nm), and axial rotation (*y*-axis, ±2.0 Nm). Loading was applied to the superior end (C2) of the vertically oriented specimen, whereas the distal portion of the cadaver (C5) remained fixed to the socket of the robot. No axial preload was used. Each test was repeated for three loading and unloading cycles at a rate of 0.2 degrees/second which approaches quasistatic testing conditions. The data from the third cycle were used for computational analysis. The tROM of the polysegment C2-5 was measured by the robot itself. To prevent desiccation during assessment, specimens were moistened with 0.9% NaCl sterile irrigation solution.

### Segmental ROM

**Figure 1:**
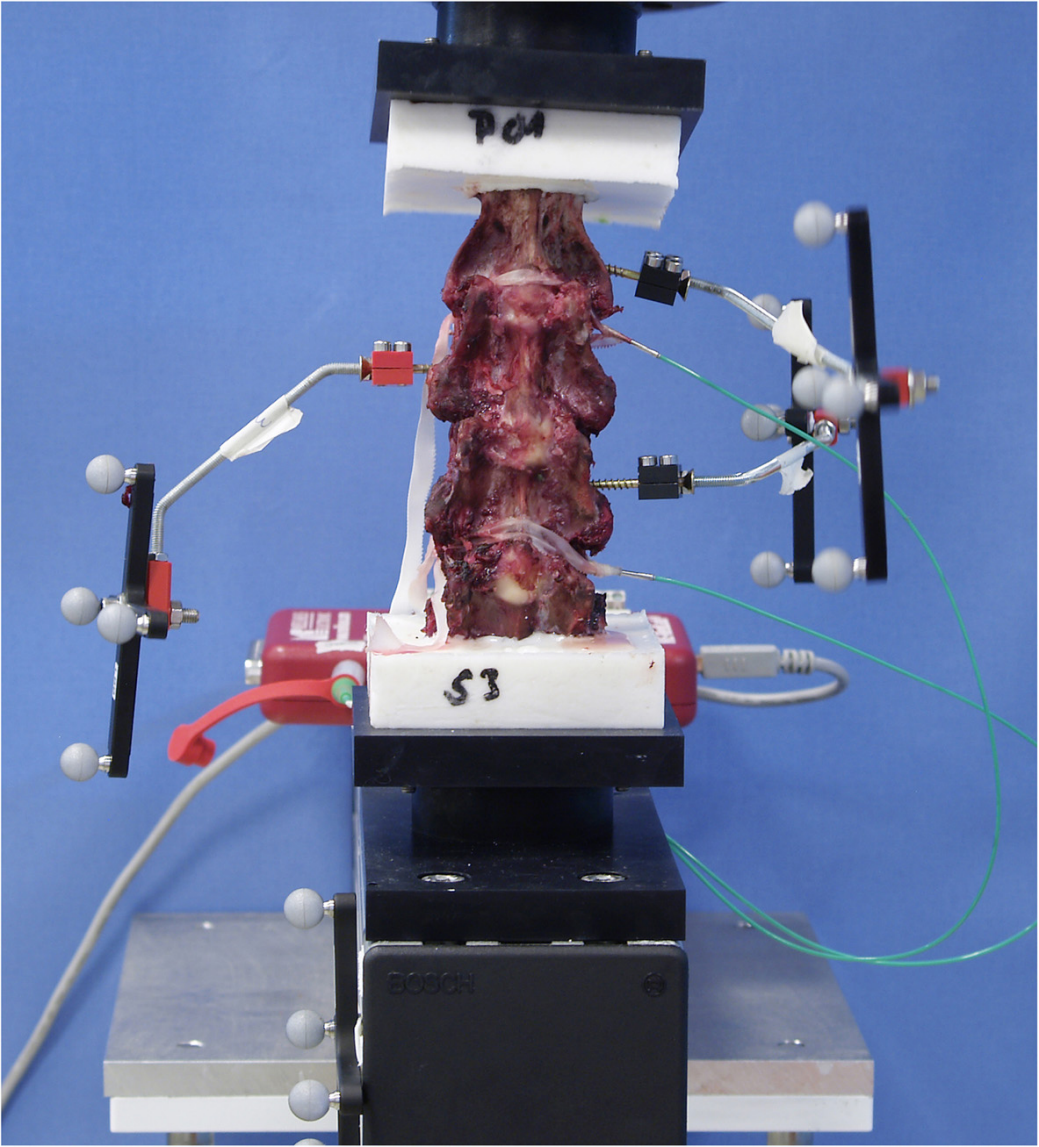
**Anterior view of an intact spine specimen.** Each rigid rod connected four optical markers to the vertebral bodies of C2 (left side), C3 (right side) and C4 (left side) for the optical tracking system, whereas the most distal tool was fixed to the socket of the robot, simulating the vertebral body of C5. The intradiscal pressure measuring sensors were inserted into the discs C2/3 and C4/5 from the left side and loosely fixed by elastic tapes to prevent dislocation.

For evaluating the iROM in the segments C2/3, C3/4 and C4/5, an optical tracking system (Polaris Northern Digital Incorporation, Ontario, Canada) was used. A rigid rod connected the tool with its four optical markers to each vertebral body of C2, C3 and C4. One tool was fixed to the socket of the robot, to which the most distal vertebral body (C5) was attached (Figure 1).

### Intradiscal pressures

Specially manufactured piezoelectric pressure measuring sensors (FMSPEZ07, MIPM GmbH, Mammendorf, Germany) were inserted into the discs of C2/3 and C4/5 from the left side (Figure 1). Their central location in the nucleus pulposus was controlled under fluoroscopy in anterior-posterior (a.p.) and lateral view. The pressure data were recorded under continuous visual control on a display. The maximum (saturation) pressure of the sensors was 7 bar.

### Reconstructive conditions

**Figure 2:**
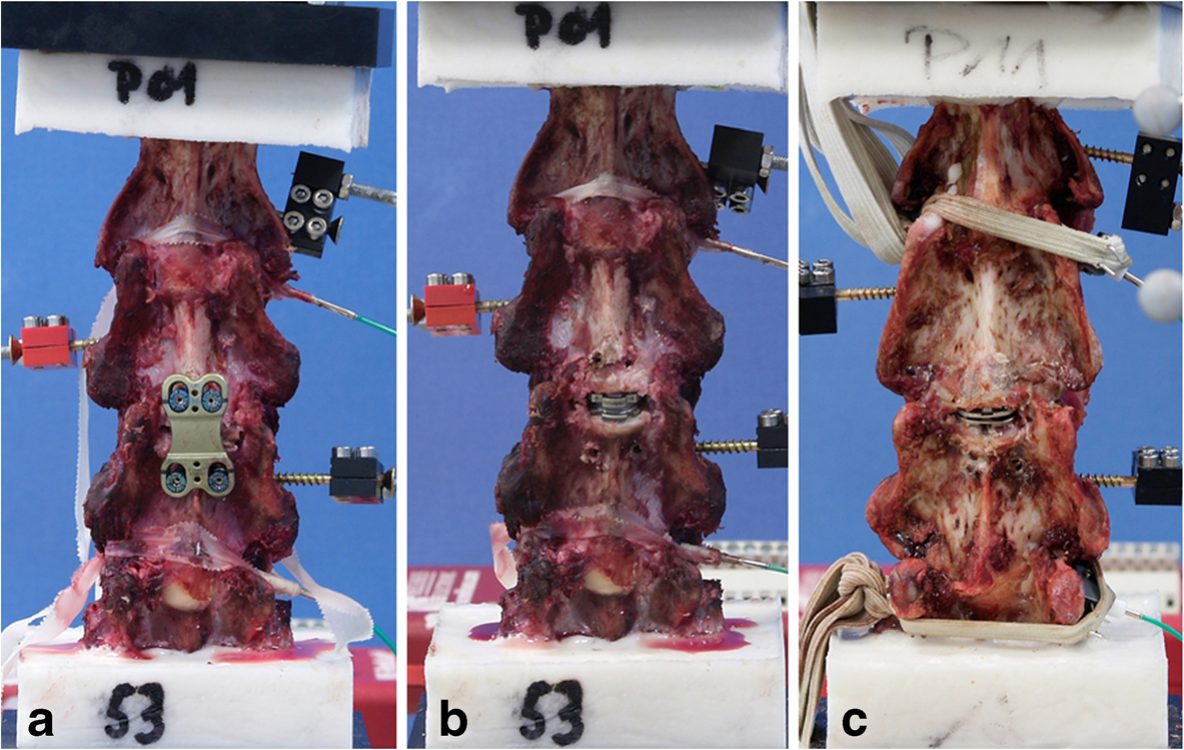
**Anterior view of the specimens in the different conditions (C2-C5): a Single-level arthrodesis C3/4 with ABC2-Plate, b single-level TDR C3/4 with Type D-prosthesis, and c single-level TDR C3/4 with Type A-prosthesis.**

The specimens were divided into two groups (group 1: specimen 01 to 06; group 2: specimen 07 to 12) and investigated in three conditions: intact, fused at the C3/4 level, and with two different types of arthroplasty, respectively as follows. After the first analysis of the intact polysegment C2-5 (group 1 and 2), a complete discectomy was performed at the level C3/4 in each specimen. The posterior longitudinal ligament (PLL) was preserved because of its pivotal role in stability [21]. Then a tricortical bone graft, which had been taken from a spinous process of the lower cervical spine, was inserted in the disc space and an anterior plate (ABC2 Anterior Cervical Plating System, Aesculap AG & Co. KG, Tuttlingen, Germany) was supplemented to simulate a single-level fusion (group 1 and 2) (Figure 2a). After testing this situation in all specimens of both groups, the plate, the screws and the bone graft were removed. Thereafter, the corresponding endplates were prepared using a high-speed burr to ensure adequate implant positioning. Cervical arthroplasty was performed according to the recommendations of the implant manufacturer. In the first six specimens (group 1), the Discover®-prosthesis (“TDR Type D”) (DePuySpine GmbH, Kirkel-Limbach, Germany) was inserted at the C3/4 level (Figure 2b), in the other six cadavers (group 2), the activ® C (“TDR Type A”) (Aesculap AG & Co. KG, Tuttlingen, Germany) was utilized as TDR, also at the C3/4 level (Figure 2c).

### Radiographic control

**Figure 3:**
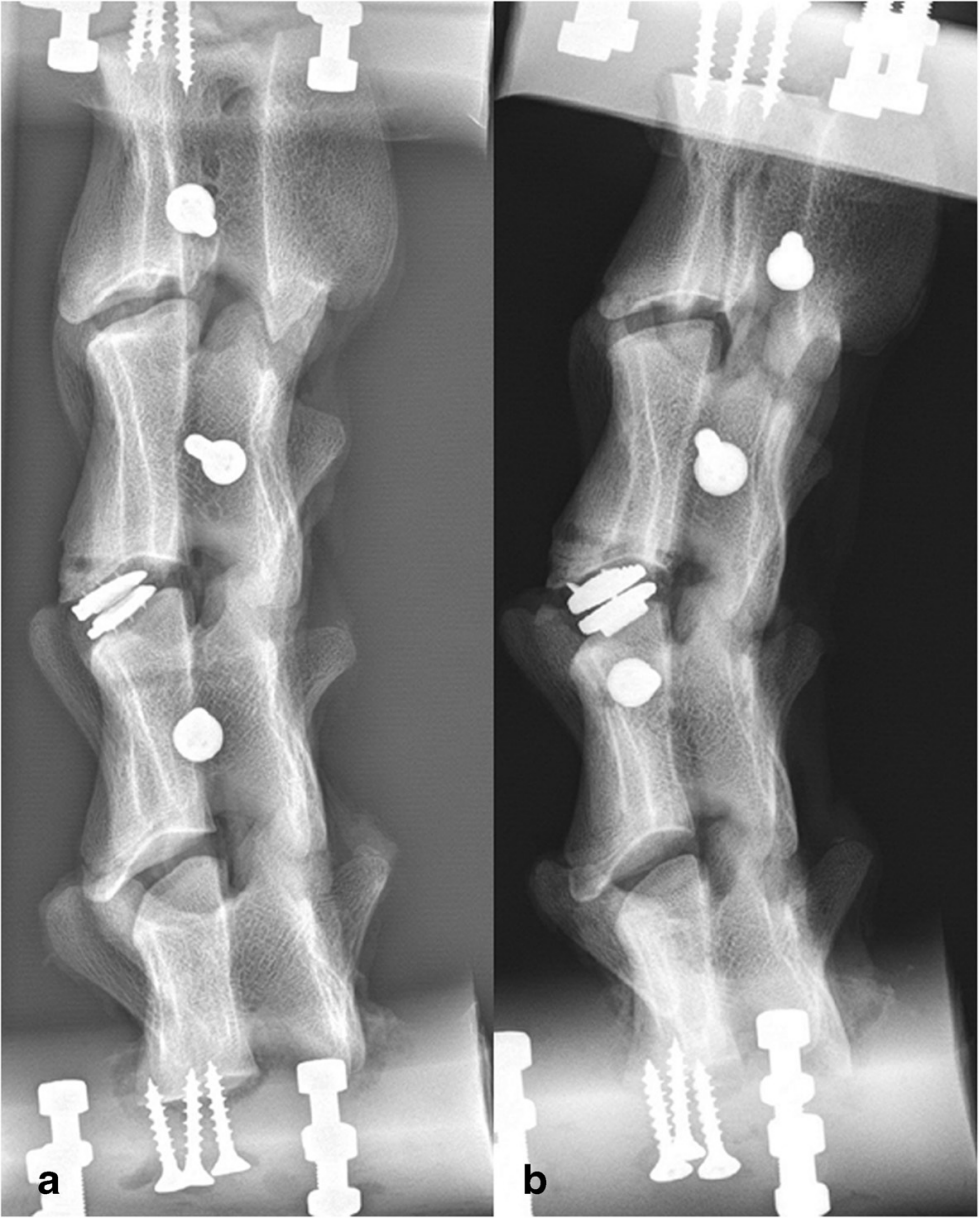
**Lateral radiograph of two different specimens.** Demonstration of the correct position of the Type D-prosthesis **(a)** and the Type A-prosthesis **(b)**.

All implanted disc prosthesis had a.p. and lateral radiographs to check their correct position in the intervertebral space (Figure 3).

### Data and statistical analysis

For statistical analysis, the data (mean value and standard deviation = SD) from the third loading cycle for the six main spinal motions were used. They were evaluated with special interest to the tROM C2-5 as the sum of the neutral and elastic zones (ROM = NZ + EZ) and representing the peak tROM (Euler angles’ rotation). The NZ is the displacement between the neutral position and the initiation point of spinal resistance to physiological motion and was calculated as well. Analogous to the tROM, the iROM at the three segments C2/3, C3/4 and C4/5 was quantified in terms of peak ROM at maximum load. The values were compared among the three different spine conditions and the percent changes in case of simulated fusion and TDR were correlated to the intact situation. The IDP in C2/3 and C4/5 in all three conditions was calculated directly to the motion angle corresponding to 90% of the value of the maximum motion angle in the fused specimen. Differences between the TDR and the other conditions (intact, arthrodesis), were compared using a paired Student’s t-test, differences between both types of the prosthesis using an unpaired Student’s t-test. A significance level of p < 0.05 was applied.

## Results

### Total ROM

**Figure 4:**
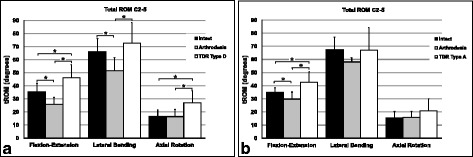
**Mean tROM [degrees] with standard deviation at the maximum loading of ±2 Nm.** Range of motion in flexion-extension, lateral bending and axial rotation in the three different conditions: intact, arthrodesis C3/4 and TDR C3/4 with Type D-prosthesis **(a)** and Type A-prosthesis **(b)**. Statistically significant differences are signed by * with bars connecting the corresponding columns.

The differences between the two groups in the intact condition were not statistically significant in all three motion directions with the following values: for flexion-extension 35.38° ±6.57 (group 1) versus 34.82° ±3.64 (group 2), for lateral bending 66.02° ±9.96 (group 1) versus 67.48° ±9.51 (group 2), and for axial rotation 16.75° ±4.77 (group 1) versus 15.25° ±5.05 (group 2). The mean tROM in flexion-extension, lateral bending and axial rotation was always recorded at the maximum loading of ±2 Nm (Figure 4a, b). In both specimen groups (group 1 and 2) we found significant different changes in flexion-extension between these conditions: intact to arthrodesis (decrease, p = 0.000 group 1; p = 0.047 group 2), arthrodesis to TDR (increase, p = 0.001 group 1; p = 0.023 group 2) and intact to TDR (increase, p = 0.005 group 1; p = 0.042 group 2). In lateral bending, only in the first six cadavers (group 1) was the difference significant between these both situations: intact to fusion (decrease, p = 0.001) and arthrodesis to TDR Type D (increase, p = 0.003). Also, in axial rotation, significant increases were seen only in the TDR Type D-group between the intact and the prosthetic condition (p = 0.017) as well as between the state of fusion and TDR (p = 0.011).

When both types of prosthesis were directly compared with each other, we found no significant differences in tROM for flexion-extension (p = 0.598), lateral bending (p = 0.652), or in axial rotation (p = 0.322).

The percentual changes of the fused condition and the state with TDR in relation to the intact situation showed in all three motion directions almost always decreasing after arthrodesis and increasing after arthroplasty in both groups (Table 1). In flexion-extension, tROM decreased by 26.68% in group 1 and by 14.19% in group 2. In lateral bending, we observed similar values with decreasing by 22.06% in group 1 and by 13.95% in group 2. In axial rotation, the changes were low with decreasing by 1.62% in group 1 and increasing by 3.08% in group 2. After implantation of the Type D-prosthesis, tROM increased by 30.77% in flexion-extension, by 9.84% in lateral bending, and by 61.96% in axial rotation, in relation to the intact condition. In the TDR Type A-group, we also observed an increasing by 22.05% in flexion-extension and by 36.67% in axial rotation, whereas in lateral bending there was a slight decreasing by 0.65%.

**Table 1:** **Changes [%] of tROM of the fused condition and the state with TDR in relation to the intact situation**

**tROM C2-5**	**Arthrodesis (group 1)**			**TDR Type D**		
	**Flexion-extension**	**Lateral bending**	**Axial rotation**	**Flexion-extension**	**Lateral bending**	**Axial rotation**
Mean	−26.68	−22.06	−1.62	30.77	9.84	61.96
SD	6.20	7.93	23.76	16.15	14.35	43.11
**tROM C2-5**	**Arthrodesis (group 2)**			**TDR Type A**		
	**Flexion-extension**	**Lateral bending**	**Axial rotation**	**Flexion-extension**	**Lateral bending**	**Axial rotation**
Mean	−14.19	−13.95	3.08	22.05	−0.65	36.67
SD	13.26	14.03	20.74	19.93	15.07	35.44

SD means standard deviation.

### Intervertebral ROM

The mean absolute values of the iROM C2/3, C3/4 and C4/5 in the intact condition increased from cranial to caudal in flexion-extension in both cadaver groups (C2/3: 8.22° and 9.52° in group 1 and group 2, C3/4: 11.74° and 11.54° in group 1 and group 2, C4/5: 14.89° and 13.55° in group 1 and group 2) (Table 2). In lateral bending, we found the following distribution in the intact state: C2/3: 21.38° (group 1) and 21.36° (group 2), C3/4: 20.89° (group 1) and 21.16° (group 2), C4/5: 23.57° (group 1) and 22.89° (group 2). In axial rotation, the corresponding values were in C2/3 5.52° and 8.11° in group 1 and group 2, in C3/4 5.44° and 7.46° in group 1 and group 2, and in C4/5: 7.69° and 6.48° in group 1 and group 2.

**Table 2:** **Values of the mean iROM [degrees] C2/3, C3/4 and C4/5 in both cadaver groups in flexion-extension, lateral bending, and axial rotation and changes [%] of the fused condition and the state with TDR in relation to the intact situation**

**iROM, F/E**	**Intact**			**Arthrodesis**			**TDR Type D**		
**Group 1**	**C2/3**	**C3/4**	**C4/5**	**C2/3**	**C3/4**	**C4/5**	**C2/3**	**C3/4**	**C4/5**
Mean	8.22	11.74	14.89	8.64	1.62*	15.88*	9.37*#	19.31*#	16.63*#
SD	2.48	1.85	2.35	2.92	1.20	2.90	3.09	4.68	2.95
Change [%]				5.06	−86.18	6.66	13.93	64.58	11.65
**iROM, LB**	**Intact**			**Arthrodesis**			**TDR Type D**		
**Group 1**	**C2/3**	**C3/4**	**C4/5**	**C2/3**	**C3/4**	**C4/5**	**C2/3**	**C3/4**	**C4/5**
Mean	21.38	20.89	23.57	21.55	4.77*	23.94	22.84	21.72#	22.84
SD	4.82	2.52	3.00	4.39	2.93	3.36	4.14	6.85	3.99
Change [%]				0.80	−77.15	1.57	6.84	3.97	−3.10
**iROM, AR**	**Intact**			**Arthrodesis**			**TDR Type D**		
**Group 1**	**C2/3**	**C3/4**	**C4/5**	**C2/3**	**C3/4**	**C4/5**	**C2/3**	**C3/4**	**C4/5**
Mean	5.52	5.44	7.69	6.36	4.59	8.52	6.31	10.93	13.26*#
SD	2.20	1.73	3.70	2.35	2.60	3.13	2.44	4.37	2.83
Change [%]				15.27	−15.50	10.77	14.32	100.95	72.31
**iROM, F/E**	**Intact**			**Arthrodesis**			**TDR Type A**		
**Group 2**	**C2/3**	**C3/4**	**C4/5**	**C2/3**	**C3/4**	**C4/5**	**C2/3**	**C3/4**	**C4/5**
Mean	9.52	11.54	13.55	10.65*	4.38*	14.76*	10.88*	16.01#	15.01*
SD	3.33	1.31	1.60	3.68	2.92	1.70	3.74	5.35	1.52
Change [%]				11.85	−62.01	8.92	14.29	38.69	10.75
**iROM, LB**	**Intact**			**Arthrodesis**			**TDR Type A**		
**Group 2**	**C2/3**	**C3/4**	**C4/5**	**C2/3**	**C3/4**	**C4/5**	**C2/3**	**C3/4**	**C4/5**
Mean	21.36	21.16	22.89	22.22	10.11*	23.87	22.89	21.00	23.53
SD	2.69	3.56	1.80	4.17	3.50	1.60	4.23	13.38	2.96
Change [%]				4.05	−52.23	4.28	7.17	−0.77	2.83
**iROM, AR**	**Intact**			**Arthrodesis**			**TDR Type A**		
**Group 2**	**C2/3**	**C3/4**	**C4/5**	**C2/3**	**C3/4**	**C4/5**	**C2/3**	**C3/4**	**C4/5**
Mean	8.11	7.46	6.48	6.37	4.45	7.06*	5.98	9.72	10.71
SD	5.12	3.12	2.45	2.40	1.55	2.01	2.32	5.30	3.94
Change [%]				−21.44	−40.31	9.03	−26.20	30.26	65.37

SD means standard deviation.

F/E = Flexion-Extension, LB = Lateral Bending, AR = Axial Rotation. *indicates significant differences to intact state, # indicates significant differences between TDR and arthrodesis.

The percentual changes of the fused condition and the state with TDR in relation to the intact situation showed significant differences in group 1 in flexion-extension iROM between arthrodesis and intact in C3/4 (decrease by 86.18%, p = 0.001) and C4/5 (increase by 6.66%, p = 0.024), between Type D-prosthesis and intact in C2/3 (increase by 13.93%, p = 0.037), C3/4 (increase by 64.58%, p = 0.007), and C4/5 (increase by 11.65%, p = 0.009) (Table 2). The increased iROM between TDR and fusion was significant in all three levels (p = 0.038 in C2/3, p = 0.001 in C3/4, p = 0.044 in C4/5). A significant decrease of lateral bending iROM by 77.15% was observed in the fused situation in C3/4 (p = 0.000), and a significant increase was seen between TDR and arthrodesis in the same segment (p = 0.002). In axial rotation, a significant increase of iROM in C4/5 had occurred only after arthroplasty by 72.31% in comparison to the intact (p = 0.012) and fused state (p = 0.032).

In comparison to the TDR Type D-group, less variations appeared in the TDR Type A-group with significant differences however observed in flexion-extension iROM between arthrodesis and intact in C2/3 (increase by 11.85%, p = 0.007), in C3/4 (decrease by 62.01%, p = 0.009), and in C4/5 (increase by 8.92%, p = 0.002). After arthroplasty, a significant increase in iROM was seen in C2/3 by 14.29%, (p = 0.011) and C4/5 by 10.75% (p = 0.032) compared to the intact cadavers. In C3/4, a significant increase was detected in relation to arthrodesis (p = 0.028). In lateral bending, the only significant change was seen in C3/4 after fusion (decrease by 52.23%, p = 0.016) and in axial rotation the only significant difference was after arthrodesis in C4/5 (increase by 9.03%, p = 0.027), always in correlation to intact.

When comparing both types of prosthesis directly, we found no significant differences in iROM of C2/3 (p = 0.616 in flexion-extension, p = 0.989 in lateral bending, p = 0.875 in axial rotation), C3/4 (p = 0.381 in flexion-extension, p = 0.919 in lateral bending, p = 0.753 in axial rotation) or C4/5 (p = 0.412 in flexion-extension, p = 0.786 in lateral bending, p = 0.369 in axial rotation).

**Figure 5:**
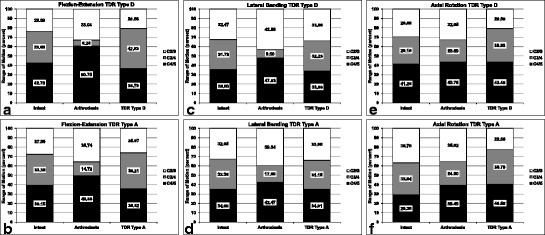
**Relative distribution of the iROM with respect to tROM for each motion segment C2/3, C3/4, and C4/5: Flexion-Extension in TDR Type D- (a) and Type A-group (b), lateral bending in Type D- (c) and Type A-group (d), and axial rotation in Type D- (e) and Type A-group (f).**

In addition to the absolute values, we analyzed the relative distribution of the iROM with respect to tROM for each motion segment C2/3, C3/4, and C4/5 (Figure 5a-f). In flexion-extension, the relative iROM decreased in the treated segment after arthrodesis in both groups (from 33.68% to 6.20% in group 1, from 33.35% to 14.72% in group 2) (Figure 5a, b). In the neighboring levels, the relative iROM always increased (in C2/3 from 23.59% to 33.04% in group 1 and from 27.50% to 35.74% in group 2, and in C4/5 from 42.73% to 60.75% in group 1 and from 39.15% to 49.55% in group 2). After implantation of both types of disc prosthesis, the relative iROM in the aim segment showed increasing with higher values compared to the intact situation (from 33.68% to 42.63% in TDR Type D-group, from 33.35% to 38.21% in TDR Type A-group). In contrast, we observed lower relative iROM in the adjacent levels compared to the physiologic state (in C2/3 from 23.59% to 20.68% in group 1 and from 27.50% to 25.97% in group 2, and in C4/5 from 42.73% to 36.70% in group 1 and from 39.15% to 35.82% in group 2). In the other two motion directions, we observed the same trend in the aim segment with decreasing of relative iROM after arthrodesis from 31.73% to 9.50% (lateral bending in group 1) and from 32.36% to 17.99% (lateral bending in group 2), as well as for axial rotation from 29.15% to 23.59% in group 1 and from 33.84% to 24.90% in group 2 (Figure 5c, d, e, f). In both neighboring levels, the relative iROM always increased after fusion, except for slight decrease in C2/3 in axial rotation in group 2 (from 36.78% to 35.62%). After arthroplasty, the relative iROM in the target segment showed always increasing in lateral bending and axial rotation compared to intact, except for slight decreasing in lateral bending from 32.36% to 31.15% in the TDR Type A-group. In each of the adjacent levels, there was no uniform pattern with some increasing and some decreasing relative values in lateral bending and axial rotation.

### Intradiscal pressure analysis

**Figure 6:**
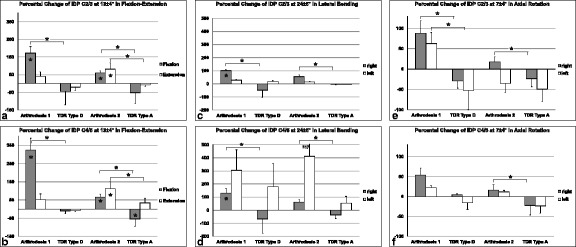
**Percentual changes of IDP C2/3 and C4/5 after arthrodesis and arthroplasty in flexion-extension (a: C2/3; b: C4/5), lateral bending (c: C2/3; d: C4/5) and axial rotation (e: C2/3; f: C4/5) in comparison to the intact condition.** The IDP was calculated directly to the motion angle (see headline) which was corresponding to 90% of the maximum value of the motion angle in the fused specimen. Statistically significant differences to intact are signed by * in the columns, significant differences between arthrodesis 1 (=group 1) and Type D- as well as arthrodesis 2 (=group 2) and Type A-prosthesis with * and bars connecting the corresponding columns.

The IDP always showed higher values in flexion and extension in the level above and below the fused segment, compared to the intact condition, which was expressed as percentual changes (Figure 6a, b). In flexion, the increasing of the IDP was always statistically significant in both discs (in C2/3 increase by 172.05% in group 1, p = 0.004 and by 59.05% in group 2, p = 0.043, in C4/5 by 324.26% in group 1, p = 0.000 and by 65.49% in group 2, p = 0.016). In extension, the IDP had also increased in the adjacent discs (in C2/3 by 41.47% in group 1 and by 81.11% in group 2, in C4/5 by 51.77% in group 1 and by 112.12% in group 2), but statistically significant only in group 2 (in C2/3 p = 0.004, in C4/5 p = 0.008). After arthroplasty, we always observed decreased IDP below the values of the intact condition in flexion in the cranial and caudal level (in C2/3 decrease by 44.30% with TDR Type D and by 51.79% with TDR Type A, in C4/5 by 10.85% with TDR Type D and by 54.50% with TDR Type A, p = 0.040). In extension, TDR led also to decreased IDP in the neighboring segments, except for C4/5 in the TDR Type A-group (increase by 33.12%).

In lateral bending, we always observed increase of IDP in C2/3 and C4/5 after arthrodesis with statistical significance in both segments to the right side in group 1 (in C2/3 increase by 101.90%, p = 0.003, in C4/5 by 130.15%, p = 0.001) (Figure 6c, d). After TDR, the changes were not significantly different compared to the intact situation, and increasing (mostly to the left side of direction) as well as decreasing (always to the right side) of the IDP had occurred.

In axial rotation, the IDP showed increased values after fusion in comparison to the physiologic state except for the motion direction to the left side in C2/3 in group 2 (decrease by 35.22%) (Figure 6e, f). All changes were not significantly different. With arthroplasty, we mostly observed decreased IDP in both groups to both directions except for a slight increase (by 4.75%) to the right side in the TDR Type D-group in C4/5. Again, the changes were not significantly different.

When comparing both types of TDR directly, we found no significant differences in the changes between the Type D- and Type A-prosthesis regarding the IDP in C2/3 (p = 0.854 in flexion, p = 0.958 in extension, p = 0.186 in lateral bending to the right side, p = 0.103 in lateral bending to the left side, p = 0.770 in axial rotation to the right side, p = 0.898 in axial rotation to the left side) and in C4/5 (p = 0.803 in flexion, p = 0.943 in extension, p = 0.219 in lateral bending to the right side, p = 0.148 in lateral bending to the left side, p = 0.312 in axial rotation to the right side, p = 0.939 in axial rotation to the left side).

## Discussion

As motion preservation technology is of great interest in the operative management of degenerative disc disease in the lumbar as well as in the cervical spine, *in vitro*-studies are important to investigate the biomechanical behavior of the different implants. In our study we tested the kinematics of polysegmental cervical cadavers from sheep under three different conditions (intact, arthrodesis and two types of semi-constrained arthroplasty). Furthermore, the pressures in the discs adjacent to the aim segment were analyzed.

As expected, total range of motion (tROM) of C2-5 decreased after simulating arthrodesis in the target segment C3/4 whereas this finding was almost without exception the clearest in the flexion-extension direction. After arthroplasty, we consistently observed an increasing tROM C2-5 in the three directions tested when compared to the intact condition, the increase was even more pronounced when contrasted to the fused situation. When comparing the data of both types of semi-constrained prosthesis, we always observed higher values of tROM with the TDR Type D than with the Type A-device. This suggests, that the Type A-prosthesis has some characteristics in its design which allow it to more closely approach the physiologic kinematic situation in the cervical spine. None the less, the differences between each of the prosthesis never reached statistical significance.

We further found increasing values of segmental ROM in the intact specimens, when progressing from the cranial to the caudal level in flexion-extension, and this progression was more distinct than in lateral bending and axial rotation. After fusion of C3/4, expectedly, the ROM in this segment always significantly decreased in flexion-extension and lateral bending in both groups. With each type of TDR, we almost always observed higher values compared to the intact state in C3/4, and with the Type D-prosthesis the relative changes were always more pronounced than with the TDR Type A. In direct comparison, the differences between both prosthesis were not statistically significant. Concerning the adjacent levels, we get the best information when analyzing the percentual distribution of the iROM in relation to the tROM, because it is independent of the absolute values of the tROM. In the intact state, we see a relative equal fraction of the percentual iROM of the three levels. After arthrodesis, the percentual iROM in the target segment C3/4 was always lowered after fusion in the three directions of motion with most reduction in flexion-extension and lateral bending in group 1 and least decreased in axial rotation in both specimen groups. Consecutively, the motion was clearly spread to each of the neighboring motion segment, most impressive in flexion-extension and lateral bending indicating a certain overload. Therefore, the percentual iROM of the neighboring levels was almost always higher (C2/3 more than C4/5) compared to the intact condition. After arthroplasty, we found always increased percentual iROM in the target level in comparison to intact state (except for lateral bending in TDR Type A-group) with percentual iROM in the adjacent segments even below the initial values, except for C2/3 in lateral bending and for C4/5 in axial rotation in both groups. These findings suggest that, from a biomechanical point of view, both semi-constrained prosthesis have influence on the target and adjacent level with leading to a certain hypermobility in the aim segments and lowering the ROM in the adjacent levels in most situations.

When we discuss our results of the IDP, we have to consider, that we did not analyze the peak values but the data which were calculated directly to the motion angle corresponding to 90% of the value of the maximum motion angle in the fused specimen. This technique became necessary because sometimes the maximum values of the IDP could not be recorded by the transducers due to extreme range of motion angles which led to pressures beyond the measuring zone, especially in the fused state. Therefore, we did not show absolute data but percentual changes of the pressure, which permits an appropriate interpretation of the results. The findings of the IDP were similar to those of the iROM in the neighboring segments. After fusion, the percentual change of the IDP was significantly higher in C2/3 and C4/5 in both specimen groups in flexion, but in extension only in the group 1, compared to the intact condition. After arthroplasty, the IDP showed decreasing in each adjacent level in flexion and extension except for extension in C4/5 with the Type A-prosthesis. In lateral bending (to the right and left side), the IDP was constantly higher in both adjacent disc spaces after simulated arthrodesis. After TDR, the IDP was lowered in C2/3 and C4/5 in lateral bending to the right side, but not significantly increased to the left side. In axial rotation and after fusion, we always observed not significantly increased IDP in C2/3 and C4/5 except for the left side in group 1. After arthroplasty, the IDP was always not significantly decreased below the initial values in both segments. We only found a not significant slight increase in C4/5 in the TDR Type D-group in axial rotation to the right side.

In summary, none of the analyzed parameters (tROM, iROM, IDP) showed any statistically significant differences between both types of prosthesis, even though the Type A-prosthesis demonstrated a more similar kinematic behavior to the intact condition than the Type D-TDR, which could eventually be an evidence of a certain degree of hypermobility of the Type D-prosthesis.

If we compare our own results with the data from other investigations, we have to consider the different study designs, which had been utilized. However, the optimal testing method has not been validated yet, so that a standardized testing protocol for analogous experiments is not available yet [22]. Nevertheless, a trend can definitely be recognized. In several experimental studies, the iROM in levels adjacent to a simulated fusion was shown to increase [5-11,13,23-25]. After TDR, the iROM in the neighboring segments approximated the corresponding values of the intact condition [5,6,8,14,23-26]. There are only two published *in vitro*-studies which investigated the kinematics after implantation of the Discover®-prosthesis in a comparable test set-up to our model [25,26]. In the presented study and the evaluation of Phillips *et al.*, the relative changes in the target segment showed significantly increasing of iROM in flexion-extension (64.6% and 43.0%) and not significantly increasing of iROM in axial rotation (101.0% and 9.1%), whereas in lateral bending we found no significant differences (4.0% and −18.9%), always compared to the intact condition [26]. Terai *et al.* recorded only in extension a 22% decrease of iROM in the treated motion segment (not significant) [25]. In flexion, lateral bending and axial rotation the changes in the iROM were similar or lower than to the intact state. Both authors did not report any significant differences of the percentual changes in the neighboring levels after implantation of the Discover®-prosthesis [25,26]. This is in contrast to our study, because in flexion-extension we found a significant higher iROM in both adjacent segments, compared to the intact cadavers, as well as in the caudal adjacent level in axial rotation. No kinematic data about the activ® C-prosthesis have been published to date. The analyses of the IDP in the present study show the same trend seen in comparable investigations with increases in the levels above and below a simulated arthrodesis [8,9,11,12]. After TDR, the values of the IDP in the adjacent segments approximate to the values of the intact situation [4,8].

Finally, the animal model and the experimental set-up are to be discussed. As already mentioned, the optimal testing method has probably not been found to date which is the conclusion of a critical review of concurrent biomechanical testing systems [22]. Probably for this reason, several different techniques to perform such experimental implant testing have been applied. Most studies utilize human cadaveric specimens, because the implants of interest are designed for clinical use in the human spine. However, we know that there are some disadvantages to human tissue testing, for instance the variability in age, height, gender, stiffness, bone quality, and grade of degenerative changes. If the goal is to perform a comparative analysis of modern motion preservation technology like TDR, it is essential to have specimens without relevant degeneration and which are of readily availability. Therefore, as others have suggested, we used the cervical spine from sheep as an accepted substitute for the human cervical spine [16-18]. When comparing our own results in the twelve ovine spines in the physiologic condition, we found a very good reproducibility of the kinematics to similar animal and human experiments. According to the study of Kandziora *et al.*, we chose the C3/4 segment as the target level for implant testing [16]. The robot used in the present study is a validated method against the Pure Moment Apparatus (PMA) and capable of applying pure, unconstrained rotational moments about three axes (*x*, *y*, *z*) [19]. Therewith, it meets all the demands of a spine tester, according to the recommendations of Wilke *et al.* [27]. One limitation of all PMA techniques is that the reference position of the specimen is force-free since it lies within the NZ of the polysegmental specimen, and is thus difficult to define. This may in part explain the asymmetrical behavior of the IDP in lateral bending and axial rotation with respect to the right versus the left side. Another declarative aspect is the fact, that we know from previous biomechanical studies with calf spines, that they behave asymmetrically [28-30]. Another possible reason for the not symmetrical distribution of the IDP to both directions in lateral bending and axial rotation can be the position of the pressure measuring sensors. Although all these transducers had been controlled by fluoroscopy in a.p. and lateral view after insertion into the disc to verify a central position in the nucleus pulposus, it cannot totally be excluded that some movement of the measuring sensors had occurred during the testing cycles even if the pressure data were recorded under continuous visual control on a display. Therefore the findings of the IDP especially in lateral bending and axial rotation should be interpreted with caution and are not be overestimated. Some investigators performed similar experimental testing with additional preload application to the specimens to imitate the influence of muscle strength [4-6,12,15,26] in contrast to the presented study, which was performed without any preload or muscle simulation analogous to previous evaluations [8,9,13,24,25]. Therefore, we showed the values for ROM and IDP not only as absolute data but also as relative changes in percentage which are expected to demonstrate the same tendency with or without any load application. However, under the influence of muscle simulation or with the use of a preload the absolute values of ROM should be decreased whereas the absolute values of IDP should be increased which is known from literature [31-33]. The applied loads in form of a pure moment with ±2.0 Nm and without an axial preload are in the lower range for an *in vitro* testing set-up for cervical spines. No damage of the bony structure or tissue was observed after testing. Therefore the range of motion of the *in vitro* tested ovine spines should be in the physiological range. As we do not know any values of the ovine spine for physical loads or for IDP *in vitro* and *in vivo* as well as for ROM *in vivo* only the relative changes in IDP after simulated fusion and arthroplasty compared to the intact state were presented and not the absolute values.

## Conclusion

The results of both the presented biomechanical investigation and similar experimental studies indicate that single-level implantation of semi-constrained TDR lead to a certain hypermobility in the treated segments with lowering the ROM in the adjacent levels in almost all situations. Nevertheless, clinical long term follow-up studies over a period of 10 to 15 years remain indispensable to observe the course *in vivo*, *i.e.* in the patient itself in order to verify, if adjacent segment pathologies are to be avoided or postponed after TDR.
